# Folie a deux: a case report

**DOI:** 10.12688/f1000research.1-18.v1

**Published:** 2012-09-27

**Authors:** Sobia Haqqi, Nisreen Ali

**Affiliations:** 1 Department of Psychiatry, Ziauddin Medical University, North Nazimabad, Karachi, Pakistan

## Abstract

Folie a deux, to date, remains a rare, yet a challenging psychiatric diagnosis. We discuss two cases that were identified in our out-patient clinics. One case was lost to follow up, while the other one showed improvement over time with appropriate management. Conclusion: As with any rare disorder, recognition and correct referral for rare diagnosis like folie a deux is of paramount importance.

## Introduction

A fascinating, rare and poorly understood psychological disorder, folie a deux, was first described by Lasègue and Falret in 1877 ^1^. It is characterized by the transference of delusions from an individual (the primary), who already suffers from a psychotic disorder, to a person or persons (the secondary) who are in close association and relative social isolation with the primary ^2^.

The relationship shared between the primary and the secondary is that of domination and submission, inducer and recipient respectively.

A review of literature showed that delusional disorders and schizophrenia were the most commonly diagnosed psychotic disorders in the primary. Delusions of persecution and grandiose accounted for most ^3^.

Family history and genetic association are considered strong factors for the development of folie a deux ^3, 4^.

Literature surrounding this disorder mainly consists of a plethora of case reports. Subsequently, it is due to these reports that there seems to be a better understanding regarding this elusive disorder.

We came across two interesting cases. In the first case, two sisters were brought in by their extended family. According to their Uncle, they were living in a one-room house with their mother who had died recently from some underlying medical illness. These three had lived in isolation for almost their entire life, with no contact with the outside world except through a domestic help to run house hold errands. On initial presentation to the psychiatry out-patient clinic, the main concern seemed to be disheveled appearance and weak physical health. Only later on, after complete psychiatric interviewing paranoid delusions against their relatives were confirmed as being shared by both. Further probing revealed that the delusions were being shared with the deceased mother. Due to some tribal reasons, the sisters were forced to leave the city and were lost to follow up.

The second case which the principal author is currently following, involves a mother and a teenage son. The mother was referred to the psychiatry out-patient clinic from the medical out-patient clinic after complete medical evaluation regarding her presenting complaints of vague aches and pains.

On initial assessment, the lady seemed guarded with her history, made no eye contact and seemed not much interested in talking about her aches and pains. She presented, almost a week later, this time accompanied with her teenage son. Shared delusions of persecution towards the husband/father were revealed on psychiatric assessment of both mother and son. The mother seemed to be the dominant psychotic individual and the son seemed to be the passive recipient.

The mother was started on atypical anti-psychotic Risperidone. The son had already plans to pursue higher studies in another city to which he was encouraged to go.

## Discussion

The primary source of knowledge regarding shared psychiatric disorders mainly consists of a plethora of case reports. This rare disorder is identified on the basis of certain established diagnostic criteria.

These include; delusion develops in an individual in the context of a close relationship with another person(s), who has an already established delusion (Criterion A), and delusions are similar in content (Criterion B), the disturbance is not accounted for by another psychotic disorder or physiological effects of a substance or any general medical condition (Criterion C) ^5^.

After a complete psychiatric anamnesis, clinical interview, psychological testing and Mental State Examination we diagnosed our patient as folie a deux.

A patient of folie a deux can present with a complaint that may be completely unrelated to his/her psychiatric condition. As reported in another case as well ^6^, our patient complained of vague aches and pains, and only after a thorough history and examination of mother and her son were we able to make a diagnosis.

According to Lazarus certain conditions have to be present for the development of folie a deux, these include an intimate emotional association between the primary and secondary and a genetic predisposition to psychosis such as blood relations with the primary case ^7^. The relationship of mother and child is usually long standing and resistant to change. This fosters an environment for folie a deux to develop as the 13 year old son; the secondary individual in this case, was predisposed through genetic and environmental factors for sharing his mother’s paranoid delusions.

The classification of shared delusional disorder by Gralnick suggests four types. *Folie impose, folie simultanée, folie communiqué, folie induite* ^8^.

Our case report is in concordance with the description of *folie impose*. Here the dominant individual, who usually suffers from a psychotic illness, imposes his/her delusion on the secondary more submissive individual and in this situation separation of the two is crucial for cure of the secondary ^8^.

As is stated through literature, the most common psychiatric disturbance that the primary suffers from is schizophrenia and the most common delusions are of persecution ^3^. In the case aforementioned the mother was diagnosed with schizophrenia, and paranoid delusions which were shared by the son.

Almost all cases in literature involve members of a nuclear family ^9^. In this 70 percent are between mother and child, husband and wife or siblings ^10^.

In these relationships the primary usually professes certain characteristics that place him/her in the position of dominance. As suggested by other case reports as well, the inducer is usually advanced age, superior intelligence with a forceful aggressive character while the induced is younger in age, paranoid, dependant and less intelligent than the primary ^11, 12^.

The relative isolation further augments the process of the transference of the delusions, and the course can usually be chronic, as there is lack of intervention and identification of the disease by other people around.

The secondary, provoked by his dependence and submissive passive personality accepts the delusions than risk losing the intimate relationship with the primary ^13^, further resulting in seclusion and separation from reality.

This type of environment breeds a mistrustful and hostile relation with the rest of the world and this further leads to paranoia which can then cause delusions of persecution as in our case.

Prognostic and therapeutic factors in folie a deux are complicated by the fact that many patients are lost to follow up. Many patients present individually which may further make identification and treatment difficult. Furthermore this relatively rare disorder has no systematic treatment regimen found to be effective. Inconsistencies in literature regarding treatment modalities exist between either one of two; Separation as the sole treatment for the secondary, or psychotherapy and medical intervention in conjunction with separation ^3^, for cure of the secondary.

Most authors concur to separation being crucial for the basis of any intervention in the management of this disorder ^14– 16^.

In our case the mother was put on atypical anti psychotics and the son was sent to live with his relatives in another city, with no pharmacological intervention. The son showed remarkable improvement with waning of the delusions and improvement in academics.

On subsequent follow up visits mother is compliant and showed no side effects of medication. Improvement was noted in her attitude towards self care with weakening of delusional ideology. However she has limited insight so far as to the nature of her psychiatric illness. Compliance and full remission is the target for now.

## Conclusion

As with any rare disorder, recognition and correct referral is of paramount importance. With timely intervention and regular follow up, Folie a deux has good prognosis. It is thus essential for psychiatrists to understand the psychodynamics of the disease and plan for long term management.
