# Supplementary material for: Folie a deux: a case report
Source: F1000Res. 2012 Sep 27;1:18. [Version 1] doi: 10.12688/f1000research.1-18.v1 (PMC3782341; doi:10.12688/f1000research.1-18.v1)
Supplement: Peer review history

# Peer review: Referee response for version 1

**Published:** 2012-10-08

**Authors:** Alfredo Carlo Altamura

**Affiliations:** 1 Department of Psychiatry, University of Milan, Milan, Italy

I have read this submission. I believe that I have an appropriate level of expertise to confirm that it is of an acceptable scientific standard.

# Peer review: Referee response for version 1

**Published:** 2012-10-01

**Authors:** Driss Moussaoui

**Affiliations:** 1 Centre Psychiatrique Universitaire, Ibn Rushd University Psychiatric Centre, Casablanca, Morocco

I read this case report and found it interesting. This kind of psychiatric pathology is mostly found in countries with strong social bonds between the members of the community, including the family.

I had a case of shared hallucinations between father and wife/daughters. When the father said “do you hear the noise?”, they said yes, and not only to please him. For women of this family, it was impossible to consider that the father may say wrong things.

Therefore, I suggest to accept this report, with a slight look at the references #2 and #5 that should be revised and completed.

I have read this submission. I believe that I have an appropriate level of expertise to confirm that it is of an acceptable scientific standard.
